# Differentiating Outcomes and Complications Between Extraplexal Tendon Transfers and Arthrodesis for Shoulder Reanimation Following Traumatic Brachial Plexus Injury: A Systematic Review and Proportional Meta-Analysis

**DOI:** 10.3390/jcm14227911

**Published:** 2025-11-07

**Authors:** Bradley J. Lauck, Jackson M. Cathey, Julian Mobley, Joshua K. Kim, Eoghan T. Hurley, Bryan S. Crook, Eliana B. Saltzman, Neill Y. Li

**Affiliations:** 1 University of North Carolina School of Medicine, Chapel Hill, NC 27599, USA; bradley_lauck@med.unc.edu (B.J.L.); julian_mobley@med.unc.edu (J.M.); 2 Duke University School of Medicine, Durham, NC 27710, USAjosh.kim@duke.edu (J.K.K.); 3 Department of Orthopaedic Surgery, Duke University School of Medicine, Durham, NC 27710, USA; 4 Division of Hand, Peripheral Nerve, and Microvascular Surgery, Department of Orthopaedic Surgery, Duke University School of Medicine, Durham, NC 27710, USA

**Keywords:** brachial plexus injury, tendon transfer, glenohumeral arthrodesis, shoulder reanimation, complications

## Abstract

**Background:** Glenohumeral arthrodesis (GHA) and extraplexal tendon transfers (TT) have been described as options for secondary shoulder stabilization and reanimation following adult traumatic brachial plexus injury (BPI) with delayed presentation or failure of primary nerve reinnervation. This study aimed to evaluate the outcomes and complication profiles of these two approaches to shoulder reanimation to better understand the indications, anticipated outcomes, and complication risks of each for traumatic brachial plexus injury. **Methods:** A systematic search of six databases (PubMed, EMBASE, SCOPUS, CINAHL, SPORTDiscus, Cochrane Library) was conducted in March 2025 following PRISMA guidelines. Studies reporting clinical outcomes in adults undergoing GHA or TT for traumatic BPI were included. Pooled mean range of motion and proportional complication and reoperation rates were calculated using random- and fixed-effects models, as appropriate. **Results:** A total of 22 studies involving 269 TT procedures and 194 GHA procedures were analyzed. Mean shoulder abduction was 81° (95% CI 54–108°) in the TT group and 51° (95% CI 37–65°) in the GHA group. Mean forward flexion was 88° (95% CI 51–124°) in the TT group and 56° (95% CI 44–68°) in the GHA group. The pooled complication rate was 4.8% (95% CI 2.6–8.6%) after TT and 26.4% (95% CI 18.5–36.1%) after GHA. The pooled reoperation rate was 3.2% (95% CI 1.5–6.6%) after TT and 17% (95% CI 10.8–25.7%) after GHA. Notably, TT cohorts generally had shorter follow-up durations, which may underrepresent late complications or reoperations. **Conclusions:** TT results in significantly lower complication and reoperation rates and demonstrates similar range-of-motion outcomes compared to GHA, suggesting that TT can be considered a first-line salvage option for motion preservation, while GHA remains an option for persistent instability, pain, or inability to achieve functional positioning of the hand in patients with traumatic BPIs. Additional comparative studies with higher levels of evidence are warranted to validate these findings.

## 1. Introduction

With a reported prevalence of 1.2% in multi-trauma patients, traumatic brachial plexus injuries (BPI) are rare but profoundly chronic and life-altering injuries [1,2]. Patients with BPI have variable upper extremity function depending on degree of injury. Nerve involvement can range from isolated upper trunk injury (C_5-6_), to upper-extended (C_5-7_), lower trunk (C_8_-T_1_) and pan-plexal injuries, all of which may result in complete loss of shoulder function [3]. Primary nerve reconstruction methods depending upon the distribution and degree of injury may provide partial to satisfactory results; however, in some cases, nerve reconstruction may not provide any return of function [4,5]. Furthermore, some BPI patterns (upper-extended, pan-plexal), may lack viable intraplexal nerve donors that can be used for reconstruction, negating the opportunity for nerve reconstruction or transfer [6].

Glenohumeral arthrodesis (GHA) and more recently tendon transfers (TT) have emerged over the past few decades as potential options for shoulder reanimation when primary reconstruction fails or for patients who present in a delayed fashion [5,7,8]. GHA provides fixed functional position of the glenohumeral joint, to facilitate improved distal function of the arm while limiting range of motion of the shoulder. Although GHA has been shown to provide acceptable functional outcomes, a significant downside to this procedure has been a higher rate of complications, including nonunion, malunion, fracture, hardware failure, and infection [9,10,11]. Conversely, TT provides an opportunity for improved shoulder range of motion through the biomechanical redistribution of existing extraplexal muscle groups. Commonly transferred tendons in BPI include upper trapezius and levator scapulae, primarily aiding in shoulder abduction, and lower trapezius, to instigate external rotation of the shoulder [5,12]. While many patients with traumatic BPI may be considered for either reanimation strategy, candidacy for each procedure depends on distinct anatomical and functional factors. TT requires viable donor musculature and adequate scapulothoracic control, whereas GHA requires sufficient bone stock and patient willingness to accept the loss of glenohumeral motion. Despite these differences, there remains limited literature to comprehensively guide clinical decision-making regarding the relative risks and benefits of these procedures.

In order to provide clearer indications, prognosis, and complication profiles between these types of shoulder reconstructions, a systematic review with meta-analysis was pursued in an effort to evaluate and quantify currently available literature. The purpose of this study was to evaluate the functional outcomes of each procedure type weighed against their complication profiles to better understand the utility of each. By doing so, this study provides greater insight into whether TT or subsets of TT provide acceptable and reliable levels of function without having to compromise on the immobility associated with fusion. We hypothesize that TT results in lower complication and reoperation rates than GHA with similar functional outcomes for patients with traumatic brachial plexus injuries.

## 2. Methods

### 2.1. Literature Search

This PROSPERO-registered systematic review (CRD42024585800) and meta-analysis was performed according to the Preferred Reporting Items for Systematic Reviews and Meta-Analyses (PRISMA) and Cochrane guidelines [13,14]. PRISMA checklist can be found in Supplementary Materials. A systematic search was performed on 13 March 2025, in the electronic databases of PubMed, Cumulative Index to Nursing and Allied Health Literature (CINAHL), EMBASE, SCOPUS, SPORTDiscus, and Cochrane Library were queried for relevant articles. The search was conducted from the inception of each database using the keywords (“brachial plexus”) AND (“arthrodesis” OR “tendon transfer”). The detailed search algorithm used for each database can be found in Supplemental Materials.

### 2.2. Study Eligibility Criteria

Peer-reviewed studies with clinical outcomes of human subjects treated with either GHA or TT for adult traumatic BPI were considered for inclusion. Studies were excluded if they consisted of interventions pertaining to obstetric BPI, iatrogenic BPI, nontraumatic BPI, or other non-traumatic pathologies for which shoulder arthrodesis or tendon transfers were performed. Additionally, we excluded systematic reviews and meta-analyses, conference abstracts, editorials, letters, animal studies, cadaveric studies, case reports, and studies lacking clinical outcome data. No restrictions were applied to manuscript language or age.

### 2.3. Screening and Data Extraction

The search results were imported into the reference management software Covidence (www.covidence.org, Veritas Health Innovation, Melbourne, Australia), where duplicates were removed. Two reviewers (B.L. and J.M.) completed the title and abstract screening. The full texts of articles that cleared these screenings were independently reviewed for final inclusion into the study. The reference lists of included studies were reviewed for additional potential inclusions. Any discrepancies were resolved by discussion with a third reviewer (J.C.) until a consensus was reached. Data was independently extracted via a standardized form. Data included information related to study design, cohort demographics, surgical interventions, functional and range of motion outcomes, complications, and reoperations where available.

### 2.4. Quality Assessment

All 22 studies included in this systematic review were non-comparative observational studies. Therefore, the Risk of Bias In Non-randomized Studies of Interventions (ROBINS-I) tool was used to perform quality assessment of the included studies and to evaluate for potential bias [15]. ROBINS-I tool rates studies as “Low”, “Moderate”, “Serious”, and “Critical” risk of bias by analyzing 7 domains including confounding bias, selection bias, bias in classification of interventions, deviation bias, missing data bias, measurement bias and reporting bias [15].

### 2.5. Statistical Analysis

Descriptive statistics are reported using totals, ranges, and percentages. Scores for continuous variables were pooled and reported as mean ± standard deviation where applicable. All statistical analysis was performed using R version 4.4.1 (R Foundation for Statistical Computing, Vienna, Austria). Given the observational nature of the included studies, a proportional meta-analysis was performed. Specifically, a meta-analysis of binomial variables was employed to analyze the prevalence of postoperative complications and reoperations among GHA and TT subgroups, using a random-effects model to account for variability both within and between studies, assuming that the true effect size may vary due to heterogeneity. Results are presented as pooled rates—weighted averages of proportions (or rates) from all included studies, accounting for their sample sizes and variances. Funnel plots were included for binomial outcome variables to visually assess for the presence of publication bias by examining the symmetry of the effect sizes across studies. A meta-analysis of continuous variables was conducted for a range of motion outcomes; given the limited number of studies reporting these data, a fixed-effects model was employed. Results are presented as pooled means. In the absence of standardized reporting of measures of variance for external rotation, Hozo’s method was applied where appropriate to generate mean and standard deviation for fixed-effect proportional meta-analysis [16]. In the absence of *p*-values to evaluate differences between groups in this methodology framework, non-overlapping 95% confidence intervals were interpreted descriptively as indicating potential differences between pooled estimates, consistent with prior proportional meta-analyses of observational studies. Significance testing was two-tailed and set at a threshold of *p* < 0.05. Heterogeneity was measured using the *I*^2^ statistic.

## 3. Results

Of the 2387 potentially eligible studies, 142 underwent full-text review, and 22 studies [4,12,17,18,19,20,21,22,23,24,25,26,27,28,29,30,31,32,33,34,35,36] were ultimately included (Supplementary Figure S1). In total, 463 procedures were included for analysis. Subgroups contained 269 procedures from 12 TT studies [4,12,17,19,20,23,25,28,30,31,32,33]. and 194 procedures from 10 GHA studies [18,21,22,24,26,27,29,34,35,36].

### 3.1. Study Characteristics and Demographics

In all studies, traumatic BPI was the primary indication for TT or GHA. In the TT cohort, 206 (91.9%) patients were male, while 146 (91.0%) were male in the GHA cohort. Regarding TT, seven studies utilized an upper trapezius transfer [17,19,28,30,31,32,33], three utilized a lower trapezius transfer [4,23,25], one utilized a combined upper and lower trapezius transfer [20], and one study utilized a levator scapulae transfer [12]. Two studies did not report patient sex [17,24]. Mean length of follow-up ranged from 6 to 29 months in the TT cohort and 28 to 238 months in the GHA cohort. The mean age at time of surgery ranged from 23.5 to 35 years in the TT cohort and 19 to 48 years in the GHA cohort. Study demographics are summarized in Table 1.

### 3.2. Risk of Bias Assessment

Quality assessment using the ROBINS-I tool revealed that all included studies demonstrated a moderate overall risk of bias. The most frequent domains contributing to this rating were confounding, reported result, and, to a lesser extent, missing data and measurement of outcomes. Across both the TT and GHA cohorts, the risk of bias related to selection, classification of interventions, and deviations from intended interventions was generally low. No studies were rated as having a serious or critical risk of bias in any domain (Supplemental Table S3).

### 3.3. Range of Motion Outcomes

**Figure 1:**
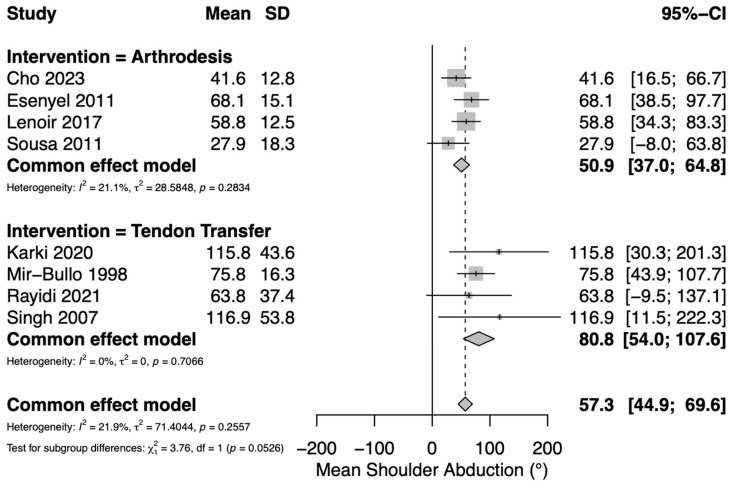
Forest plot depicting pooled mean postoperative shoulder abduction (reported in degrees) between interventions [22,27,29,34]. Tendon transfers were either upper trapezius (Karki [28], Mir-Bullo [30], Singh [33]) or levator scapulae (Rayidi) [12].

Mean postoperative active shoulder abduction was reported in nine studies: four assessing TT [12,28,30,33], and four assessing GHA [22,27,29,34]. Three TT studies utilized the upper trapezius [28,30,33] and one utilized the levator scapulae [12]. A pooled meta-analysis of continuous variables yielded an overall mean postoperative active shoulder abduction of 57.3° (95% CI 45–70°) for both groups. Mean postoperative active shoulder abduction in the TT group was 81° (95% CI 54–108°) and mean postoperative active shoulder abduction in the GHA group was 51° (95% CI 37–65°) (Figure 1). Low heterogeneity was observed among the included studies (*I*^2^ = 21.9%).

**Figure 2:**
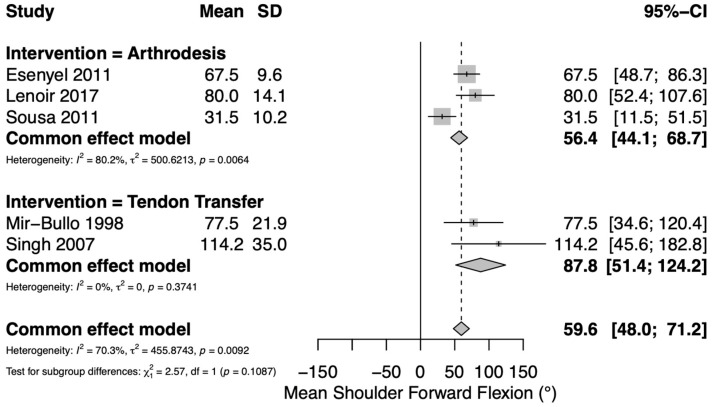
Forest plot depicting pooled postoperative shoulder forward flexion (reported in degrees) between interventions. Both tendon transfer studies utilized an upper trapezius transfer [27,29,30,33,34].

Mean postoperative active shoulder forward flexion was reported in six studies: two assessing TT [30,33] and three assessing GHA [27,29,34]. Both TT studies utilized an upper trapezius transfer. A pooled meta-analysis of continuous variables yielded an overall mean postoperative active shoulder forward flexion of 60° (95% CI 48–71°). Mean postoperative shoulder forward flexion in the TT group was 88° (95% CI 51–124°) and mean postoperative shoulder forward flexion in the GHA group was 56° (95% CI 44–69°) (Figure 2). Substantial heterogeneity was observed among the included studies (*I*^2^ = 70.3%). Range of motion outcomes by type of tendon transfer are summarized in Supplementary Table S2.

**Figure 3:**
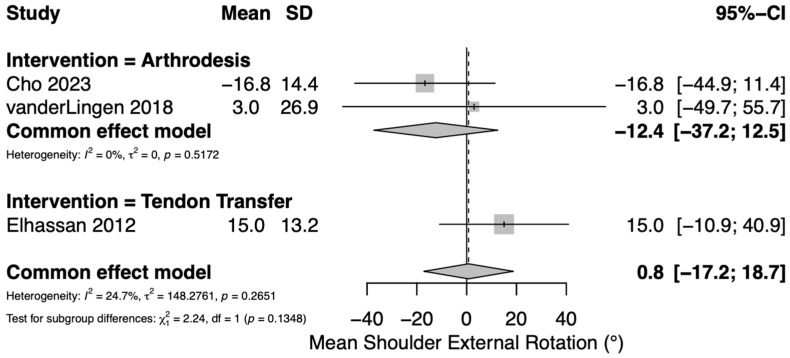
Forest plot depicting pooled mean postoperative shoulder external rotation (reported in degrees) between interventions. Tendon transfers were all from the lower trapezius [4,22,36].

Postoperative active shoulder external rotation was reported in five studies: three assessing TT [4,23,25] and two assessing GHA [22,36]. All three TT studies utilized a lower trapezius transfer. We were unable to include two TT studies in our meta-analysis due to the absence of central tendency or variance measures in these studies [23,25]. For TT, shoulder external rotation results are presented in Supplementary Table S2. Postoperative means for shoulder external rotation after TT ranged from 15 to 50°. For GHA, a pooled meta-analysis of continuous variables yielded an overall mean postoperative active shoulder external rotation of −12° (95% CI −37–13°) (Figure 3). Cho et al. found a mean −13.5° of external rotation after GHA in a cohort of 15 patients [22], while van der Lingen et al. reported a mean of 0° (range −58–30°) in a cohort of 12 patients [36].

### 3.4. Patient-Reported Outcomes

Postoperative patient-reported outcomes (PROs) were reported in eight studies: four assessing TT [4,23,25,28] and four assessing GHA [27,29,35,36]. There was substantial heterogeneity among the reporting systems used, which limited the ability to conduct a comparative analysis. The most commonly reported PROs were the Disabilities of the Arm, Shoulder and Hand (DASH) (n = 6) [4,23,25,28,29,36], Visual Analog Scale (VAS) (n = 3) [4,27,36], and the Subjective Shoulder Value (SSV) (n = 3) [4,25,35]. The American Shoulder and Elbow Score (ASES) and Simple Shoulder Test (SST) were reported in only one GHA study [29]. Patient-reported outcomes are summarized in Supplementary Table S3, where available.

### 3.5. Complications

**Figure 4:**
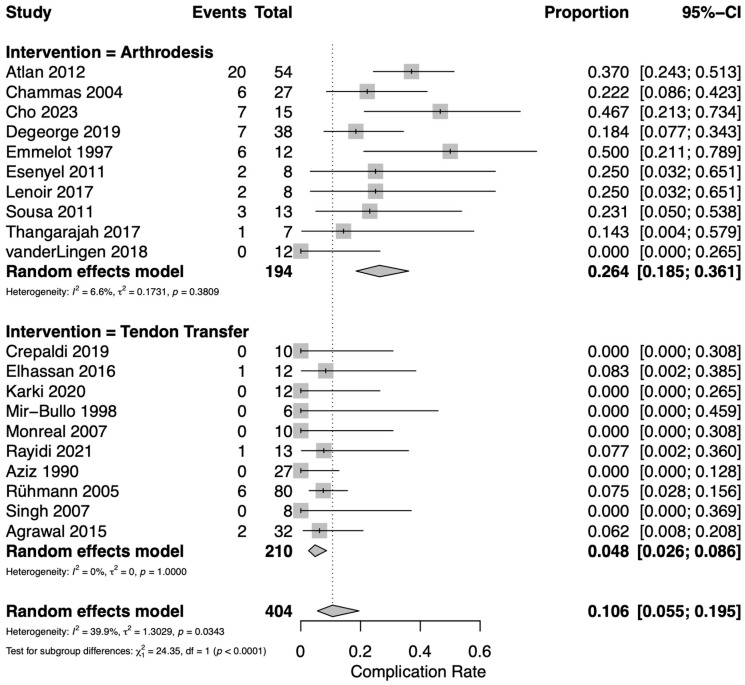
Forest plot for pooled proportion estimates of complication rate between interventions [12,17,18,19,21,22,23,24,25,26,27,28,29,30,31,32,33,34,35,36].

Complication rates were reported in 20 studies: 10 assessing TT [12,17,19,23,25,28,30,31,32,33] and 10 assessing GHA [18,21,22,24,26,27,29,34,35,36]. The overall pooled complication rate including both TT and GHA was 10.6% (95% CI 5.5–19.5%). TT resulted in significantly fewer complications than GHA (Figure 4). For TT, the pooled complication rate was 4.8% (95% CI 2.6–8.6%). Among the pooled TT data, 10 complications were observed including 4 cases of infection, 2 humeral fractures, 2 cases of hardware loosening, 1 graft retear, and 1 hematoma. For GHA, the pooled complication rate was 26.4% (95% CI 18.5–36.1%). Among the pooled GHA data, 54 complications were observed including 20 cases of nonunion, 18 humeral fractures, 9 cases of infection, 5 cases of symptomatic hardware, 1 malunion, and 1 scapular fracture. Moderate heterogeneity was observed among the included studies (*I*^2^ = 39.9%). The funnel plot for data included in the proportional meta-analysis of complications does not demonstrate asymmetry which would suggest publication bias (Supplementary Figure S2). It is important to note that TT cohorts were typically followed for less than two years, whereas several glenohumeral arthrodesis series included long-term follow-up. It is possible this discrepancy may contribute to lower observed complication and reoperation rates in the TT group, as late mechanical failures are more likely to be captured in the longer arthrodesis series.

### 3.6. Reoperations

**Figure 5:**
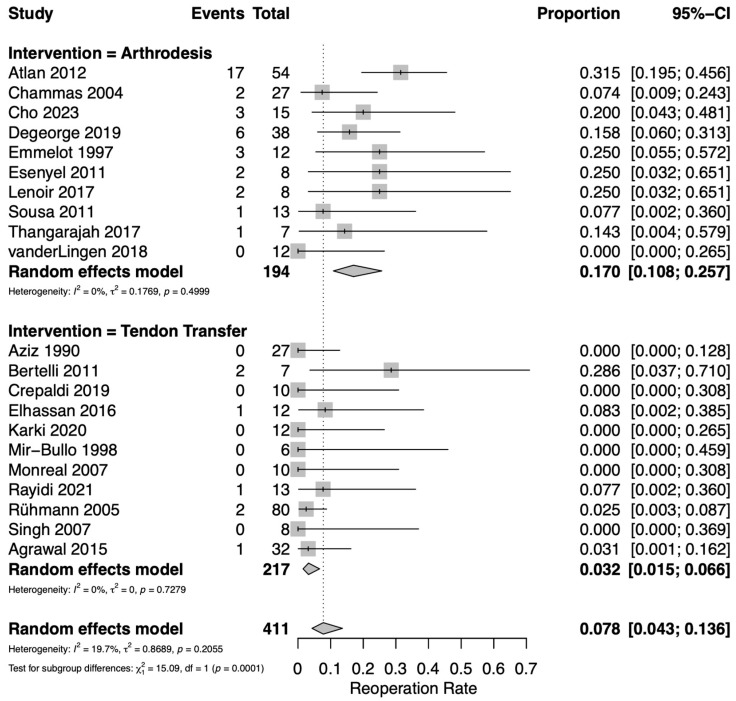
Forest plot for pooled proportion estimates of reoperation rate between interventions [12,17,18,19,20,21,22,23,24,25,26,27,28,29,30,31,32,33,34,35,36].

Reoperation rates were reported in 21 studies: 11 assessing TT [12,17,19,20,23,25,28,30,31,32,33] and 10 assessing GHA [18,21,22,24,26,27,29,34,35,36]. The overall pooled rate of reoperation including both TT and GHA was 7.8% (95% CI 4.3–13.6%). TT resulted in significantly fewer reoperations than GHA (Figure 5). For TT, the pooled reoperation rate was 3.2% (95% CI 1.5–6.6%). Among the pooled TT data, 7 reoperations were observed including 3 cases of symptomatic hardware removal, 1 for revision TT, 2 for conversion to arthrodesis, and 1 hematoma evacuation. For GHA, the pooled reoperation rate was 17.0% (95% CI 10.8–25.7%). Among the pooled GHA data, 37 reoperations were observed including 20 cases for revision arthrodesis, 10 for symptomatic hardware removal, 6 for humeral open reduction and internal fixation, and 1 irrigation and debridement. Low heterogeneity was observed among the included studies (*I*^2^ = 19.7%). The funnel plot for data included in the proportional meta-analysis of complications does not demonstrate asymmetry which would suggest publication bias (Supplementary Figure S3).

## 4. Discussion

Traumatic BPI can result in significant functional deficits, particularly affecting mobility and stability of the glenohumeral joint which may have a significant impact on quality of life. This is the first study in the literature to systematically compare the outcomes of TT and GHA for shoulder reanimation following traumatic BPI. The primary finding of this study was that TT offers favorable functional outcomes and is associated with a markedly lower complication and reoperation rate when compared to GHA. While both procedures improved shoulder function, there were no statistically significant differences were found with respect to postoperative ROM. However, considerable heterogeneity among study designs, surgical techniques, and reporting metrics was observed, which should be considered when interpreting the pooled results.

The tendon transfer studies included in this review utilized at least one of the upper trapezius, lower trapezius, or levator scapulae tendons to improve range of motion and shoulder function after traumatic BPI. Restoration of shoulder external rotation and abduction are the most important planes of movement to improve function and quality of life in patients with a disabled limb [37]. The upper trapezius transfer is historically the most common tendon transfer performed for traumatic BPI and was used in over half of the studies in our review. Transfer of this tendon has provided good outcomes in the literature, especially with improving stability and shoulder abduction/flexion, but it is not useful in addressing a lack of shoulder external rotation [5,37]. Conversely, transfer of the lower trapezius is known to produce the most substantial gain in shoulder external rotation and has become an increasingly popular option to treat traumatic BPI. It is important to note that many treatment plans may include the transfer of multiple tendons for maximal functional improvement such as upper and lower trapezius. Given the variability in tendon availability and injury patterns among patients with traumatic BPI, individualized surgical planning is crucial to optimize functional recovery and improvement.

In our study, both TT and GHA demonstrated measurable improvements in shoulder function, though range of motion outcomes were inconsistently reported across studies. While our analysis did not find statistically significant differences, it is possible that the observed trends favoring TT may reflect the limitations of the small sample size rather than lack of true effect. The TT group had a 30° greater pooled estimate for shoulder abduction, although this difference did not reach statistical significance. Notably, these studies utilized an upper trapezius transfer technique which offers the biomechanical advantage of primarily restoring abductive function [4]. Although we were unable to perform comparative meta-analyses for shoulder external rotation, transfers involving the lower trapezius demonstrated around 25° of motion which provides useful function. Regarding PROs, the most commonly reported among the two procedures was DASH, which revealed similar outcomes. Although PROs were too heterogeneous for meta-analysis, all included studies reported postoperative improvement, and scores such as DASH and SSV were consistently within functional ranges. The survey time point was inconsistent among studies, as some reported postoperative PROs, while others reported preoperative or only pre- to postoperative changes in scores. Standardization of outcome measures and consistent reporting criteria are imperative for future studies to more definitively and translationally understand the differences in clinical outcomes of these procedures.

Our analysis also demonstrated TT resulted in substantially lower complication and reoperation rates compared to GHA. The most common postoperative complications after TT were infection and graft tear that commonly occur in the early postoperative period. Interestingly, a recent review of TT for treatment of massive rotator cuff tears reported higher complications compared to our findings, but the majority were also from early postoperative issues such as hematomas or surgical site infections [38]. The rates of other common TT complications such as graft retear and hardware loosening were similar to those found in our analysis [38]. In contrast, GHA was associated with a higher complication rate with the majority of complications being either nonunion or humeral fractures. While humeral fractures usually respond well to treatment, nonunion is a complication that poses significant challenges and may persist despite revision, bone-grafting, and other additional procedures [39]. Moreover, the reoperation rates observed in this study also align with prior literature, with TT demonstrating significantly lower rates compared to GHA [5,9]. Generally, the complications seen after GHA were more severe compared to the complications seen after TT (nonunion, malunion, humeral fracture), which highlights its potential to confer significant long-term morbidity and healthcare resource utilization.

Several limitations were identified in this study. First, the paucity of available literature, primarily case series and retrospective cohort studies, restricted the robustness of our analysis. Although we utilized strict exclusion criteria, it is plausible the true complication and reoperation rates may be higher than reported due to the preferential publishing of outcomes. Given the level of heterogeneity of the included studies, random-effect models were employed for all meta-analyses, which may have restricted our ability to detect significant differences in ROM outcomes. We were unable to include postoperative external rotation in our meta-analysis due to heterogeneity in reporting methods. Similarly, significant heterogeneity existed in PROs reported for each study, which therefore precluded meaningful comparative analyses. Furthermore, we noted substantial differences in preoperative injury characteristics, such as injury severity, preoperative function, and brachial plexus injury patterns, complicating the interpretation of our comparisons. Additionally, the follow-up durations differed between TT and GHA cohorts, which may have influenced the observed complication and reoperation rates. However, this effect is likely minimal, as prior studies indicate that most TT complications occur early in the postoperative period [38]. Finally, the studies included in this review employed variable surgical techniques for TT and GHA, which we were unable to account for. These limitations underscore the profound need for comparative studies with aligned and consistent patient-reported and objective outcome measures, to elucidate the differences in outcomes between these two procedures for traumatic BPI.

## 5. Conclusions

This systematic review and proportional meta-analysis revealed that tendon transfer results in significantly lower complication and reoperation rates and demonstrates similar, or in some cases, superior range of motion outcomes compared to glenohumeral arthrodesis. These findings suggest that when viable donor musculature is present, tendon transfer can be considered a first-line salvage option for motion preservation, while glenohumeral arthrodesis remains an option for persistent instability, pain, or inability to achieve functional positioning of the hand. Additional comparative studies with higher levels of evidence and coordinated efforts in patient injury patterns, techniques, and outcome measures are needed to best assess long-term outcome differences between these two approaches to shoulder stability and reanimation.

## Figures and Tables

**Table 1:** Demographics of All Included Studies by Study Type.

					Age (Years)		FU (Months)		Age of Lesion (Mean, Range)	Type of Tendon Transfer
Authors	Year	LOE	N	Male, %	Mean	Range	Mean	Range		
**Arthrodesis**										
Atlan [18]	2012	3	54	100	24	18–51	42	3–81	31 (7–57)	
Chammas [21]	2004	3	27	93	25	17–37	71		33	
Cho [22]	2023	4	15	93	31.6	20–47	40.9	10–94		
Degeorge [24]	2019	3	38		30	18–60	58.1	24–188		
Emmelot [26]	1997	3	12	83	19	16–26	84	6–108	32	
Esenyel [27]	2011	4	8	88	39.3	22–68	66.6	47–96		
Lenoir [29]	2017	4	8	100	33	26–58	28	12–40	46 (21–93)	
Sousa [34]	2011	4	13	85	46	35–68	101	13–149		
Thangarajah [35]	2017	4	7	86	48	28–80	98	27–197	60 (30–96)	
van der Lingen [36]	2018	4	12	92	46	27–61	238			
**Tendon Transfer**										
Agrawal [17]	2015	2	32		23.5	17–42	8.25			Upper trap
Aziz [19]	1990	4	27	85	31.2	14–58	14.6	6–46	31.3 (6–120)	Upper trap
Bertelli [20]	2011	4	7	100	27.6	21–41	11.7	6–36		Upper + lower trap
Crepaldi [23]	2019	2	10	100	24		6		15.1	Lower trap
Elhassan [4]	2012	4	52	79	27	20–69	19	12–28		Lower trap
Elhassan [25]	2016	4	12	83	35	19–59	23	12–37	19	Lower trap
Karki [28]	2020	4	12	100	26.7	21–34	6		24 (14–36)	Upper trap
Mir-Bullo [30]	1998	4	6	100	28	20–42	19	12–25	31 (12–57)	Upper trap
Monreal [31]	2007	4	10	80	28.3	17–41	17.5	16–52	37.2 (14–75)	Upper trap
Rayidi [12]	2021	4	13	100	30	17–47	12			Levator scapulae
Rühmann [32]	2005	4	80	86	31	18–69	28.8	9.6–96	73.2 (9.6–444)	Upper trap
Singh [33]	2007	4	8	88	31.5	18–50	15.3	10–24	20.3 (11–36)	Upper trap

LOE, Level of evidence; FU, Follow-up period; Age of lesion defined as time from injury to index surgery.

## Data Availability

Extracted data available upon request made to corresponding author.

## Supplementary Materials

The following supporting information can be downloaded at: https://www.mdpi.com/article/10.3390/jcm14227911/s1.

## References

[1] Kaiser R., Waldauf P., Ullas G., Krajcova A. (2020). Epidemiology, etiology, and types of severe adult brachial plexus injuries requiring surgical repair: Systematic review and meta-analysis. Neurosurg. Rev..

[2] Kokkalis Z., Papagiannis S., Kouzelis A., Diamantakis G., Panagopoulos A. (2022). Traumatic Bilateral Brachial Plexus Injury. Cureus.

[3] Hems T.E. (2015). Timing of surgical reconstruction for closed traumatic injury to the supraclavicular brachial plexus. J. Hand Surg. Eur. Vol..

[4] Elhassan B., Bishop A.T., Hartzler R.U., Shin A.Y., Spinner R.J. (2012). Tendon transfer options about the shoulder in patients with brachial plexus injury. J. Bone Jt. Surg..

[5] Zhang D., Garg R., Earp B.E., Blazar P., Dyer G.S.M. (2021). Shoulder Arthrodesis versus Upper Trapezius Tran.sfer for Traumatic Brachial Plexus Injury: A Proportional Meta-Analysis. Adv. Orthop..

[6] Li N.Y., Wu K.Y., Loosbrock M.F., Bishop A.T., Spinner R.J., Shin A.Y. (2024). Injury and Biological Factors Impact Shoulder Function following Autogenous Grafting of Spinal Nerves for Pan-Brachial Plexus Reconstruction. Plast. Reconstr. Surg..

[7] Hermena S., Assaf A., Donaldson O. (2021). Systematic Review With Meta-Analysis: Are Muscle Transfers a Satisfactory Treatment Option to Restore Shoulder Abduction in Delayed Adult Brachial Plexus Injuries?. Cureus.

[8] Noland S.S., Bishop A.T., Spinner R.J., Shin A.Y. (2019). Adult Traumatic Brachial Plexus Injuries. J. Am. Acad. Orthop. Surg..

[9] Adu-Kwarteng K., Cabell G.H., Hurley E.T., Amanah A.Y., Levin J.M., Lassiter T.E., Boachie-Adjei Y.D., Klifto C.S., Anakwenze O. (2024). Glenohumeral arthrodesis outcomes and complications: A systematic review. J. Shoulder Elb. Surg..

[10] Sobhi S., Bochat K., Booth G., Mattin A., Moniz S. (2024). Clinical and Surgical Outcomes of Shoulder Arthrodesis. J. Clin. Med..

[11] Wagner E.R., McLaughlin R., Sarfani S., Cofield R.H., Sperling J.W., Sanchez-Sotelo J., Elhassan B.T. (2018). Long-Term Outcomes of Glenohumeral Arthrodesis. J. Bone Jt. Surg..

[12] Rayidi V.K.R., R S., Appaka J. (2021). Functional Evaluation of Levator Scapulae Tendon to Supraspinatus in Adult Brachial Plexus Injuries. Indian J. Plast. Surg..

[13] Cumpston M., Li T., Page M.J., Chandler J., Welch V.A., Higgins J.P., Thomas J. (2019). Updated guidance for trusted systematic reviews: A new edition of the Cochrane Handbook for Systematic Reviews of Interventions. Cochrane Database Syst. Rev..

[14] Page M.J., McKenzie J.E., Bossuyt P.M., Boutron I., Hoffmann T.C., Mulrow C.D., Shamseer L., Tetzlaff J.M., Akl E.A., Brennan S.E. (2021). The PRISMA 2020 statement: An updated guideline for reporting systematic reviews. BMJ.

[15] Sterne J.A.C., Hernán M.A., Reeves B.C., Savović J., Berkman N.D., Viswanathan M., Henry D., Altman D.G., Ansari M.T., Boutron I. (2016). ROBINS-I: A tool for assessing risk of bias in non-randomised studies of interventions. BMJ.

[16] Wan X., Wang W., Liu J., Tong T. (2014). Estimating the sample mean and standard deviation from the sample size, median, range and/or interquartile range. BMC Med. Res. Methodol..

[17] Agrawal N.K. (2015). Transfer of upper trapezius with clavicular segment for restoration of shoulder movements following injury to the brachial plexus. Plast. Aesthetic Res..

[18] Atlan F., Durand S., Fox M., Levy P., Belkheyar Z., Oberlin C. (2012). Functional outcome of glenohumeral fusion in brachial plexus palsy: A report of 54 cases. J. Hand Surg..

[19] Aziz W., Singer R.M., Wolff T.W. (1990). Transfer of the trapezius for flail shoulder after brachial plexus injury. J. Bone Jt. Surg. Br..

[20] Bertelli J.A. (2011). Upper and lower trapezius muscle transfer to restore shoulder abduction and external rotation in longstanding upper type palsies of the brachial plexus in adults. Microsurgery.

[21] Chammas M., Goubier J.N., Coulet B., Reckendorf G.M., Picot M.C., Allieu Y. (2004). Glenohumeral arthrodesis in upper and total brachial plexus palsy. A comparison of functional results. J. Bone Jt. Surg. Br..

[22] Cho A.B., Choi H.J., Ferreira C.H.V., Yoshinobu Kiyohara L., Bersani Silva G., Sorrenti L. (2023). Shoulder Arthrodesis for Traumatic Brachial Plexus Injuries: Functional Outcomes and Complications. Hand.

[23] Crepaldi B.E., Neto J.Q.L., Rezende M.R., Júnior R.M., Scarcella D.S. (2019). Lower Trapezius Transfer for Patients With Brachial Plexus Injury. Hand.

[24] Degeorge B., Lazerges C., Chammas P.E., Coulet B., Lacombe F., Chammas M. (2019). Comparison of spinal accessory nerve transfer to supra-scapular nerve vs. shoulder arthrodesis in adults with brachial plexus injury. Orthop. Traumatol. Surg. Res..

[25] Elhassan B.T., Wagner E.R., Spinner R.J., Bishop A.T., Shin A.Y. (2016). Contralateral Trapezius Transfer to Restore Shoulder External Rotation Following Adult Brachial Plexus Injury. J. Hand Surg..

[26] Emmelot C.H., Nielsen H.K., Eisma W.H. (1997). Shoulder fusion for paralyzed upper limb. Clin. Orthop. Relat. Res..

[27] Esenyel C.Z., Oztürk K., Imren Y., Ayanoğlu S. (2011). Shoulder arthrodesis with plate fixation. Acta Orthop. Traumatol. Turc..

[28] Karki D., Muthukumar V., Dash S., Singh A.K. (2020). Trapezius Transfer to Restore Shoulder Function in Traumatic Brachial Plexus Injury: Revisited and Modified. J. Hand Surg. Asian-Pac. Vol..

[29] Lenoir H., Williams T., Griffart A., Lazerges C., Chammas M., Coulet B., Le Nen D. (2017). Arthroscopic arthrodesis of the shoulder in brachial plexus palsy. J. Shoulder Elb. Surg..

[30] Mir-Bullo X., Hinarejos P., Mir-Batlle P., Busquets R., Carrera L., Navarro A. (1998). Trapezius transfer for shoulder paralysis. 6 patients with brachial plexus injuries followed for 1 year. Acta Orthop. Scand..

[31] Monreal R., Paredes L., Diaz H., Leon P. (2007). Trapezius transfer to treat flail shoulder after brachial plexus palsy. J. Brachial Plex. Peripher. Nerve Inj..

[32] Rühmann O., Schmolke S., Bohnsack M., Carls J., Wirth C.J. (2005). Trapezius transfer in brachial plexus palsy. J. Bone Jt. Surg. Ser. B.

[33] Singh A., Karki D. (2007). Modified trapezius transfer technique for restoration of shoulder abduction in brachial plexus injury. Indian J. Plast. Surg..

[34] Sousa R., Pereira A., Massada M., Trigueiros M., Lemos R., Silva C. (2011). Shoulder arthrodesis in adult brachial plexus injury: What is the optimal position?. J. Hand Surg. Eur. Vol..

[35] Thangarajah T., Lambert S.M. (2017). Glenohumeral arthrodesis for late reconstruction of flail shoulder in patients with traumatic supraclavicular brachial plexus palsy. Shoulder Elb..

[36] van der Lingen M.A.J., de Joode S., Schotanus M.G.M., Grimm B., van Nie F.A., Speth L., Samijo S.K. (2018). Satisfied patients after shoulder arthrodesis for brachial plexus lesions even after 20 years of follow-up. Eur. J. Orthop. Surg. Traumatol..

[37] Elhassan B., Bishop A., Shin A., Spinner R. (2010). Shoulder tendon transfer options for adult patients with brachial plexus injury. J. Hand Surg..

[38] Desai V., Stambulic T., Daneshvar P., Bicknell R.T. (2023). Lower trapezius tendon transfer for irreparable rotator cuff injuries: A scoping review. JSES Rev. Rep. Tech..

[39] Scalise J.J., Iannotti J.P. (2008). Glenohumeral arthrodesis after failed prosthetic shoulder arthroplasty. J. Bone Jt. Surg..

