# Supplementary material for: Differentiating Outcomes and Complications Between Extraplexal Tendon Transfers and Arthrodesis for Shoulder Reanimation Following Traumatic Brachial Plexus Injury: A Systematic Review and Proportional Meta-Analysis
Source: J Clin Med. 2025 Nov 7;14(22):7911. doi: 10.3390/jcm14227911 (PMC12653131; doi:10.3390/jcm14227911)
Supplement: Supplementary file 1 [file jcm-14-07911-s001.zip › jcm-3957833-supplementary/searchstrategy.pdf]

Database Search Strategies

Pubmed
915 results

("Brachial Plexus"[Mesh] OR "brachial plexus" [TIAB] OR bpi[tiab] OR (plexus [TIAB] AND brachial*[TIAB]) OR "plexus brachialis"[tiab]) AND (Brachial Plexus/injuries[mesh] OR brachial plexus/surgery[mesh] OR injur*[tiab] OR damag*[tiab] OR degenerat*[tiab] OR impair*[tiab] OR trauma*[tiab] OR denervat*[tiab] OR neuropath*[tiab] OR nerve*[tiab] OR wounds and injuries[mesh] OR surg*[tiab] OR surgery[mesh]) AND (tendon transfer[mesh] OR "Tendon transfer*"[tiab] OR "muscle transfer*" OR "tendon transfer"[title/abstract:~3] OR "muscle transfer"[title/abstract:~3] OR "shoulder arthrodesis"[title/abstract:~3] OR ((shoulder joint[mesh] OR glenohumeral[tiab] OR shoulder*[tiab]) AND (arthrodesis[mesh]) OR arthrodes*[tiab] OR fusion*[tiab])) NOT (case reports[pt] OR comment[pt] OR letter[pt] OR editorial[pt])

EMBASE
1264 results

('brachial plexus'/exp OR 'brachial plexus':ti,ab OR bpi:ti,ab OR (plexus:ti,ab AND brachial*:ti,ab) OR 'plexus brachialis':ti,ab) AND ('nerve injury'/exp OR 'brachial plexus injury'/exp OR 'injury'/exp OR injur*:ti,ab OR damag*:ti,ab OR degenerat*:ti,ab OR impair*:ti,ab OR trauma*:ti,ab OR denervat*:ti,ab OR neuropath*:ti,ab OR nerve*:ti,ab OR wound*:ti,ab OR surg*:ti,ab OR 'surgery'/exp) AND ('tendon transfer'/exp OR 'muscle transplantation'/exp OR 'shoulder arthrodesis'/exp OR 'tendon transfer*':ti,ab OR 'muscle transfer*' OR 'shoulder arthrodes*':ti,ab OR (('shoulder joint'/exp OR 'shoulder'/exp OR glenohumeral:ti,ab OR shoulder*:ti,ab) AND ('arthrodesis'/exp OR arthrodes*:ti,ab OR fusion*:ti,ab)))

CINAHL
122 Results

(((MH "Brachial Plexus+" OR TI "brachial plexus" OR AB "brachial plexus" OR TI bpi OR AB bpi OR ((TI plexus OR AB plexus) AND (TI brachial* OR AB brachial*)) OR TI "plexus brachialis" OR AB "plexus brachialis")) AND ((TI injur* OR AB injur* OR TI damag* OR AB damag* OR TI degenerat* OR AB degenerat* OR TI impair* OR AB impair* OR TI trauma* OR AB trauma* OR TI denervat* OR AB denervat* OR TI neuropath* OR AB neuropath* OR TI nerve* OR AB nerve* OR TI wound* OR AB wound* OR TI injur* OR AB injur* OR TI surg* OR AB surg* OR MH surgery+ OR MH “wounds and injuries+” OR MH “brachial plexus neuropathies+”))) AND (((MH "shoulder injuries+" OR TI glenohumeral OR AB glenohumeral OR TI shoulder* OR AB shoulder*) AND (MH arthrodesis+ OR TI arthrodesis OR AB arthrodesis OR TI fusion* OR AB fusion*)) OR (TI "Tendon transfer*" OR AB "Tendon transfer*" OR TI "muscle transfer*" OR AB "muscle transfer" OR MH arthrodesis+" OR TI arthrodesis OR AB arthrodesis))

SCOPUS
1125 results

(INDEXTERMS ("Brachial Plexus") OR TITLE-ABS ("brachial plexus") OR TITLE-ABS (bpi) OR (TITLE-ABS (plexus) AND TITLE-ABS (brachial*)) OR TITLE-ABS ("plexus brachialis")) AND (INDEXTERMS ("Brachial Plexus") OR INDEXTERMS ("brachial plexus") OR TITLE-ABS (injur*) OR TITLE-ABS (damag*) OR TITLE-ABS (degenerat*) OR TITLE-ABS (impair*) OR TITLE-ABS (

trauma*) OR TITLE-ABS (denervat*) OR TITLE-ABS (neuropath*) OR TITLE-ABS (nerve*) OR wounds AND INDEXTERMS (injuries) OR TITLE-ABS (surg*) OR INDEXTERMS (surgery)) AND (INDEXTERMS ("tendon transfer") OR TITLE-ABS ("Tendon transfer*") OR "muscle transfer*" OR INDEXTERMS ("tendon transfer") OR INDEXTERMS ("muscle transfer") OR INDEXTERMS ("shoulder arthrodesis") OR ((INDEXTERMS ("shoulder joint") OR TITLE-ABS (glenohumeral) OR TITLE-ABS (shoulder*)) AND (INDEXTERMS (arthrodesis)) OR TITLE-ABS (arthrodes*) OR TITLE-ABS (fusion*))) AND (EXCLUDE (DOCTYPE , "ed") OR EXCLUDE (DOCTYPE , "le") OR EXCLUDE (DOCTYPE , "no") OR EXCLUDE (DOCTYPE , "ch"))

SportDiscus
414 results

(DE "Brachial Plexus" OR TI "brachial plexus" OR AB "brachial plexus" OR TI bpi OR AB bpi OR TI "plexus brachialis" OR AB "plexus brachialis") AND (TI injur* OR AB injur* OR TI damag* OR AB damag* OR TI degenerat* OR AB degenerat* OR TI impair* OR AB impair* OR TI trauma* OR AB trauma* OR TI denervat*" OR AB "denervat*" OR TI neuropath* OR AB neuropath* OR TI nerve* OR AB nerve* OR TI surg* OR AB surg* OR DE "surgery") AND (TI "Tendon transfer*" OR AB "Tendon transfer*" OR "muscle transfer*" OR AB "muscle transfer*" OR DE arthrodesis OR ((DE "shoulder+” OR DE “shoulder pain+” OR TI glenohumeral OR AB glenohumeral OR TI shoulder* OR AB shoulder*) AND (DE "arthrodesis+" OR TI arthrodes* OR AB arthrodes* OR TI fusion* OR AB fusion*)))

Cochrane Library
13 results

(Brachial NEXT Plexus OR bpi OR plexus NEXT brachialis) AND (injur* OR damag* OR degenerat* OR impair* OR trauma* OR denervat* OR neuropath* OR nerve* OR surg* OR surgery) AND (tendon NEXT transfer* OR muscle NEXT transfer* OR arthrodesis OR ((shoulder* OR glenohumeral) AND (arthrodesis OR fusion*)))
