# Supplementary material for: Differentiating Outcomes and Complications Between Extraplexal Tendon Transfers and Arthrodesis for Shoulder Reanimation Following Traumatic Brachial Plexus Injury: A Systematic Review and Proportional Meta-Analysis
Source: J Clin Med. 2025 Nov 7;14(22):7911. doi: 10.3390/jcm14227911 (PMC12653131; doi:10.3390/jcm14227911)
Supplement: Supplementary file 1 [file jcm-14-07911-s001.zip › jcm-3957833-supplementary/Supp Table 1.pdf]

**Table S1.** ROBINS-I

| Study | Year | Cohort | Confounding | Selection | Classification of Intervention | Deviation from Intended Intervention | Missing Data | Measurement of Outcomes | Reported Result | Overall |
|---|---|---|---|---|---|---|---|---|---|---|
| Atlan | 2012 | GHA | Moderate | Low | Low | Low | Low | Low | Moderate | Moderate |
| Chammas | 2004 | GHA | Moderate | Low | Low | Low | Low | Low | Low | Moderate |
| Cho | 2023 | GHA | Moderate | Low | Low | Low | Low | Low | Moderate | Moderate |
| Degeorge | 2019 | GHA | Moderate | Low | Low | Low | Moderate | Low | Low | Moderate |
| Emmelot | 1997 | GHA | Moderate | Low | Moderate | Low | Low | Moderate | Moderate | Moderate |
| Esenyel | 2011 | GHA | Moderate | Low | Low | Low | Moderate | Moderate | Moderate | Moderate |
| Lenoir | 2017 | GHA | Moderate | Low | Low | Low | Moderate | Low | Low | Moderate |
| Sousa | 2011 | GHA | Moderate | Low | Low | Low | Moderate | Low | Low | Moderate |
| Thangarajah | 2017 | GHA | Moderate | Low | Low | Low | Low | Low | Low | Moderate |
| van der Lingen | 2018 | GHA | Moderate | Low | Low | Low | Low | Low | Moderate | Moderate |
| Crepaldi | 2019 | TT | Moderate | Low | Low | Low | Low | Low | Moderate | Moderate |
| Elhassan | 2012 | TT | Moderate | Low | Low | Low | Low | Low | Low | Moderate |
| Elhassan | 2016 | TT | Moderate | Low | Moderate | Low | Low | Low | Low | Moderate |
| Aziz | 1990 | TT | Moderate | Low | Low | Low | Low | Moderate | Moderate | Moderate |
| Karki | 2020 | TT | Moderate | Low | Low | Low | Low | Low | Low | Moderate |
| Mir-Bullo | 1998 | TT | Moderate | Low | Low | Low | Low | Low | Low | Moderate |
| Monreal | 2007 | TT | Moderate | Low | Low | Low | Low | Low | Low | Moderate |
| Rayidi | 2021 | TT | Moderate | Low | Moderate | Low | Low | Low | Low | Moderate |
| Rühmann | 2005 | TT | Moderate | Low | Low | Low | Low | Low | Low | Moderate |
| Singh | 2007 | TT | Moderate | Low | Low | Low | Low | Low | Low | Moderate |
| Agrawal | 2015 | TT | Moderate | Low | Low | Low | Low | Low | Low | Moderate |
| Bertelli | 2011 | TT | Moderate | Low | Low | Low | Low | Moderate | Moderate | Moderate |

GHA, Glenohumeral arthrodesis; TT, Tendon transfer.
