# Supplementary material for: Differentiating Outcomes and Complications Between Extraplexal Tendon Transfers and Arthrodesis for Shoulder Reanimation Following Traumatic Brachial Plexus Injury: A Systematic Review and Proportional Meta-Analysis
Source: J Clin Med. 2025 Nov 7;14(22):7911. doi: 10.3390/jcm14227911 (PMC12653131; doi:10.3390/jcm14227911)
Supplement: Supplementary file 1 [file jcm-14-07911-s001.zip › jcm-3957833-supplementary/Supp Table 2.pdf]

Table S2. Summary of postoperative range of motion in degrees grouped by the primary tendon used for transfer.

	Abduction		Forward flexion		External rotation	
**Upper Trapezius**	**Mean**	**Range**	**Mean**	**Range**	**Mean**	**Range**
Agrawal	85	45-140	55	40-90		
Aziz*	45.4	20-120	35.2	0-120		
Karki	116	10-180				
Mir-Bullo	76	50-100	78	45-110		
Monreal*	46.2		37.4			
Rühmann	34	5-90	30	5-90		
Singh	116	45-180	107	90-180		
**Lower Trapezius**						
Crepaldi	56		57		15	
Elhassan	60	40-100	50	40-70	20	-20-40
Elhassan					50	
**Upper + Lower Trapezius**						
Bertelli	38					
**Levator scapulae**						
Rayidi	60					

*Values presented as pre to postoperative change in range of motion (degrees).
