# Supplementary material for: Differentiating Outcomes and Complications Between Extraplexal Tendon Transfers and Arthrodesis for Shoulder Reanimation Following Traumatic Brachial Plexus Injury: A Systematic Review and Proportional Meta-Analysis
Source: J Clin Med. 2025 Nov 7;14(22):7911. doi: 10.3390/jcm14227911 (PMC12653131; doi:10.3390/jcm14227911)
Supplement: Supplementary file 1 [file jcm-14-07911-s001.zip › jcm-3957833-supplementary/Supp Table 3.pdf]

Table S3. Summary of patient-reported outcomes at final follow-up.

	Year	Tendon Transferred	DASH	VAS	SSV	ASES	SST
**Arthrodesis**							
Esenyel	2011			2.9 (1-7)			
Lenoir	2017		36			69	4
Thangarajah	2017				45 (15-100)		
van der Lingen	2018		15 (8-46)	49 (0-96)			
**Tendon Transfer**							
Crepaldi	2019	Lower trap	26.7				
Elhassan	2012	Lower trap	7	2 (1-3)	40		
Elhassan	2016	Lower trap	37		45		
Karki	2020	Upper trap	38				

Values are presented as mean (range); DASH, Disabilities of the Arm, Shoulder and Hand score; VAS, Visual Analog Scale score; SSV, Subjective Shoulder Value; ASES, American Shoulder and Elbow Surgeons score; SST, Simple Shoulder Test score.
