# Supplementary figures and images for: Differentiating Outcomes and Complications Between Extraplexal Tendon Transfers and Arthrodesis for Shoulder Reanimation Following Traumatic Brachial Plexus Injury: A Systematic Review and Proportional Meta-Analysis
Source: J Clin Med. 2025 Nov 7;14(22):7911. doi: 10.3390/jcm14227911 (PMC12653131; doi:10.3390/jcm14227911)

# Complications

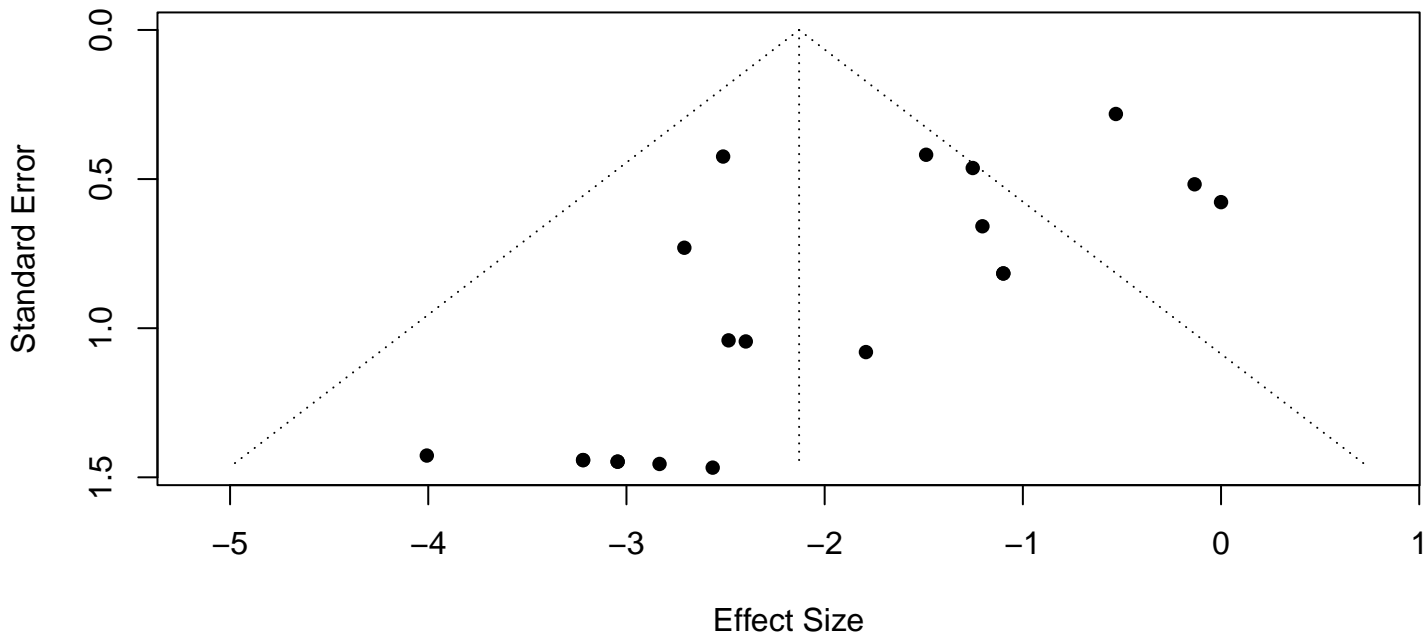

Supplement: Supplementary file 1 [file jcm-14-07911-s001.zip › jcm-3957833-supplementary/Funnel_Comp_supp fig 2.pdf]

## Reoperations

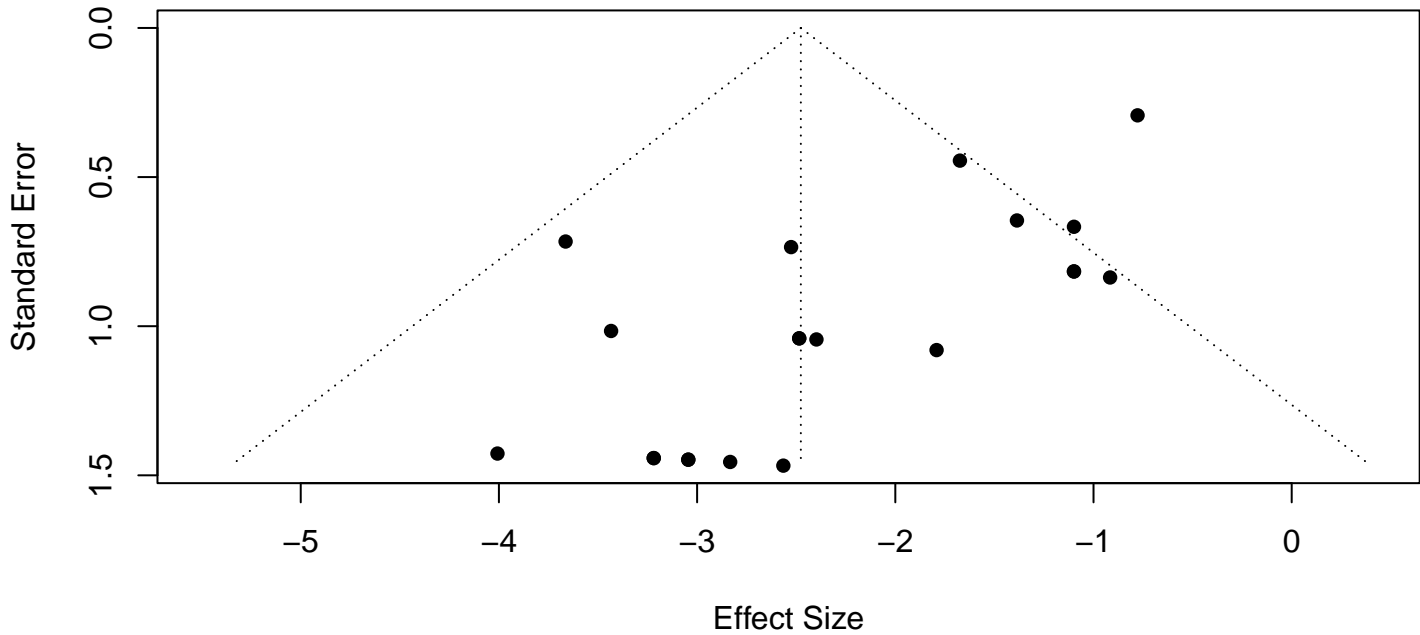

Supplement: Supplementary file 1 [file jcm-14-07911-s001.zip › jcm-3957833-supplementary/Funnel_Reop_supp fig 3.pdf]

## Supplemental Figures

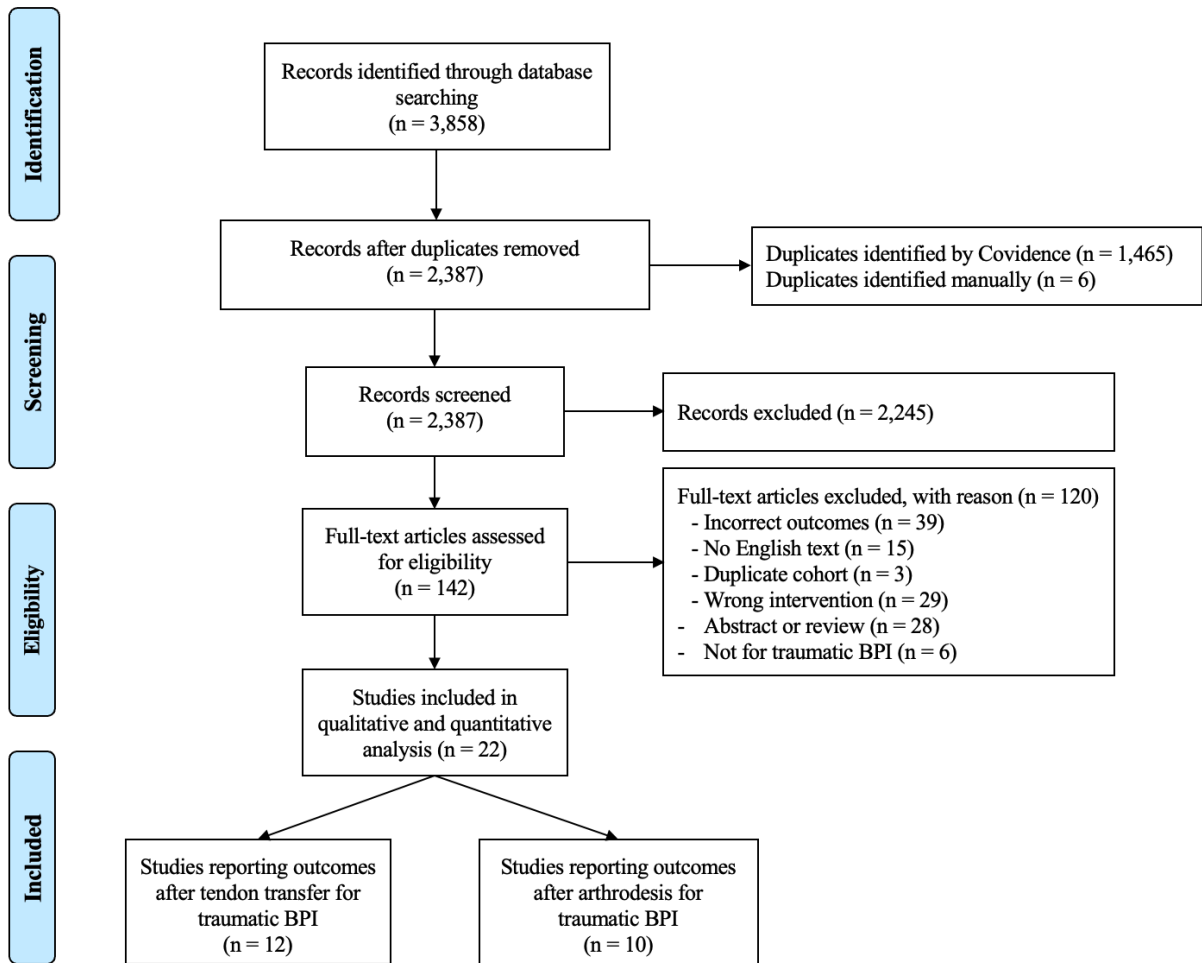

Figure S1. PRISMA Chart.

Supplement: Supplementary file 1 [file jcm-14-07911-s001.zip › jcm-3957833-supplementary/Supp figure 1.pdf]
